# Blood Monocyte Subsets and Selected Cardiovascular Risk Markers in Rheumatoid Arthritis of Short Duration in relation to Disease Activity

**DOI:** 10.1155/2014/736853

**Published:** 2014-07-14

**Authors:** Ewa Klimek, Tomasz Mikołajczyk, Joanna Sulicka, Beata Kwaśny-Krochin, Mariusz Korkosz, Grzegorz Osmenda, Barbara Wizner, Andrzej Surdacki, Tomasz Guzik, Tomasz K. Grodzicki, Anna Skalska

**Affiliations:** ^1^Department of Internal Medicine and Gerontology, Jagiellonian University Medical College, University Hospital Śniadeckich Street 10, 31-531 Cracow, Poland; ^2^Department of Internal and Agricultural Medicine, Jagiellonian University Medical College, J. Dietl Hospital, Skarbowa Street 4, 31-121 Cracow, Poland; ^3^Department of Rheumatology and Balneology, Jagiellonian University Medical College, University Hospital, Śniadeckich Street 10, 31-531 Cracow, Poland; ^4^Division of Rheumatology, Department of Internal Medicine and Gerontology, Jagiellonian University Medical College, University Hospital, Śniadeckich Street 10, 31-531 Cracow, Poland; ^5^2nd Department of Cardiology, Jagiellonian University Medical College, University Hospital, Kopernika Street 17, 31-501 Cracow, Poland

## Abstract

*Objectives*. To evaluate blood monocyte subsets and functional monocyte properties in patients with rheumatoid arthritis (RA) of short duration in the context of cardiovascular (CV) risk and disease activity. *Methods*. We studied conventional markers of CV risk, intima media thickness (IMT), and blood monocyte subsets in 27 patients aged 41 ± 10 years with RA of short duration (median 12 months) and 22 healthy controls. The RA subjects were divided into low (DAS28: 2.6–5.1) and high (DAS28 > 5.1) disease activity. *Results*. RA patients exhibited increased levels of intermediate (CD14^++^CD16^+^) monocytes with decreased CD45RA expression compared to controls, increased counts of classical (CD14^++^CD16^−^) monocytes, and decreased percentages of nonclassical (CD14^+^CD16^++^) monocytes. Patients with high disease activity had lower HLA DR expression on classical monocytes compared to low disease activity patients. There were no differences in monocyte subsets between subjects with DAS > 5.1 and DAS ≤ 5.1. There were no significant intergroup differences in IMT and the majority of classical CV risk factors. *Conclusions*. Patients with RA of short duration show alteration in peripheral blood monocyte subsets despite the fact that there is no evidence of subclinical atherosclerosis. Disease activity assessed with DAS28 was associated with impaired functional properties but not with a shift in monocyte subpopulations.

## 1. Introduction

Monocytes/macrophages play an important role in the pathogenesis of rheumatoid arthritis (RA) [1], disease associated with accelerated atherogenesis [2, 3]. They migrate into joints and settle in the synovium and at the cartilage-pannus junction, where they sustain inflammation and promote proliferation of the synovium and joint destruction in both the acute and the chronic phase of RA [1]. Activation of monocytes is reflected by expression of major histocompatibility complex (MHC) class II surface receptor—HLA-DR [4] and by expression of antigen CD45RA [5]. Monocytes produce proinflammatory cytokines such as interleukin- (IL-) 1*β*, IL-6, and tumor necrosis factor alpha (TNF-*α*) [6]. These cytokines are released into the systemic circulation resulting in endothelial activation/dysfunction [7] preceding the formation of atherosclerotic lesion. *β*_2_-integrins (CD11c/CD18, CD11b/CD18) are expressed on monocytes and their ligands (intercellular adhesion molecule-1 (ICAM-1), vascular cell adhesion molecule-1 (VCAM-1))—on activated endothelium [8, 9].

Due to increased integrin overexpression monocytes adhere to activated endothelium and migrate into the arterial wall, where foam cells arise from monocyte-derived macrophages to form fatty streaks.

A key indicator of early vascular changes in the process of atherosclerosis is an increased intima media thickness (IMT) of the common carotid artery [10, 11]. It was shown previously that RA patients have signs of subclinical atherosclerosis, expressed by increased carotid IMT, even without traditional CV risk factors [12] and an active disease was independently associated with increased IMT [12, 13]. It has also been established in other groups of patients without overt cardiovascular disease that increased IMT heralds increased risk for cardiac events and stroke [14].

Human monocytes consist of two major subpopulations, classical (CD14^++^CD16^−^) monocytes constituting 80–90% of blood monocytes and CD16^+^ monocytes, which are subdivided into two subsets: intermediate (CD14^++^CD16^+^) and nonclassical (CD14^+^CD16^++^) monocytes [15]. Furthermore, there appears to be a developmental relationship between monocyte subsets—from classical via intermediate to nonclassical [15]. Undeniably monocytes play important role in the pathogenesis of both RA [1] and atherosclerosis [16]. However, it has not been established so far which subset of monocytes is mainly involved in these processes. In the study of Heine et al. [17], elevated level of intermediate monocytes correlated with increased risk of ischemic cardiovascular disease in end stage renal disease. Increased prevalence of CD16^+^ monocytes has been reported by Kawanaka et al. in the peripheral circulation of patients with active RA [18], but without distinguishing between their subpopulations. Rossol et al. pointed to a role and an expansion of the intermediate (CD14^++^CD16^+^) monocyte population in patients with RA without growth within the population of nonclassical (CD14^+^CD16^+^) monocytes [19]. Similar conclusions were reached by Cooper et al. [20]. However, all the above studies focused on patients with long-standing RA.

To the best of our knowledge, peripheral blood monocytes heterogeneity has not been extensively studied in patients with RA of short duration so far. Therefore, our goal was to evaluate peripheral blood monocyte subpopulations and their functional properties and to assess traditional CV risk factors and signs of subclinical atherosclerosis in patients with RA of short duration in relation to disease activity.

## 2. Methods

### 2.1. Study Population

We studied 27 adult patients, with RA of short duration recruited from the group of patients with arthritis referred to the Outpatient Rheumatology Clinic of the Department of Internal Diseases of the University Hospital in Krakow as published previously [21], two patients were excluded due to lack of flow cytometry results. The diagnosis of RA was established according to the revised 1987 American College of Rheumatology (formerly, the American Rheumatism Association) criteria [22]. The patients have had disease duration of ≥6 weeks, have not been treated with any biological or nonbiological disease-modifying antirheumatic drugs (DMARDs), have been without any treatment (4 patients), or have been receiving stabile dose of nonsteroidal anti-inflammatory drugs (NSAIDs) and/or steroids at least 4 weeks prior to enrollment in our study. Treatment with DMARDs was given to all patients with diagnosis of RA according to current guidelines directly after completion of the study protocol. For all RA patients, DAS28 including high-sensitivity C-reactive protein (hsCRP), the tender joint count (28 joints), the swollen joint count (28 joints), and the patient's assessment of global well-being (100 mm visual analogue scale, VAS) was calculated [23]. We divided patients with RA according to DAS28 into two groups: with high (DAS28 > 5.1) and low (DAS28: 2.6–5.1) disease activity. Three patients with DAS28 below 2.6 were excluded from the subgroup analysis. Additionally, we studied 22 healthy control subjects matched for sex and age.

Exclusion criteria, common for both groups, included clinical evidence of atherosclerotic CV disease (coronary artery disease, history of acute coronary syndrome, stroke/transient ischemic attack, peripheral artery disease, and symptomatic carotid artery stenosis), uncontrolled/untreated hypertension, diabetes, renal failure, chronic or acute infection, other autoimmune diseases, a history of neoplastic disease within 5 years after treatment termination, and current therapy with DMARDs.

### 2.2. Study Protocol

Patients were recruited from July 2009 to June 2011. The procedure was carried out in the morning in the outpatient clinic. The subjects had been previously asked to refrain from eating, smoking and alcohol, or caffeine consumption for at least 12 h. All desired data, mainly on smoking status, accompanying diseases and taking drugs were collected according to prepared earlier questionnaire. Blood pressure was measured 3 times on the left arm after 5 minutes of rest in a sitting position; values from 2 last readings were averaged. Mean arterial pressure (MAP) was calculated using the following equation: [diastolic blood pressure + 1/3 (systolic blood pressure − diastolic blood pressure)]. Anthropometric measures including weight and height were taken and body mass index (BMI) (body mass [kg]/height [m]^2^) was calculated. Then all participants underwent blood sampling for biochemical assays and flow cytometry. The study protocol was approved by the Bioethical Committee of Jagiellonian University and written informed consent was obtained from all participants [21].

### 2.3. Biochemical Assays

Blood samples were taken from the left antecubital vein. Serum lipids [total cholesterol (TC), LDL-cholesterol (LDL-C), HDL-cholesterol (HDL-C), triglycerides (TG)] and glucose levels were measured with a Hitachi 917 analyzer (Roche Diagnostics, Hitachi Ltd., Japan) using standardized laboratory techniques. High-sensitivity C-reactive protein (hsCRP) was measured with immunonephelometry (Nephelometer BM II, Siemens Health Care Diagnostics Inc., USA).

In RA patients, rheumatoid factor (RF) was determined by immunoturbidimetric assay (APTEC Diagnostics nv., ALLmed Diagnostics) and anticyclic citrullinated peptide antibodies (aCCP) with ELISA (QUANTA Lite CCP 3.1 IgG/IgA ELISA, INOVA Diagnostics, Inc., San Diego, USA).

### 2.4. Flow Cytometry

As described previously [24], peripheral blood mononuclear cells (PBMC) were isolated on the day of collection from peripheral EDTA-anticoagulated blood by density gradient centrifugation with LSM 1077 (Lymphocyte Separation Medium, PAA Laboratories GmbH, Austria). Cells (100.000) were stained for 20 minutes on ice with fluorochrome-conjugated monoclonal antibodies (anti-CD14-APC-H7, anti-CD16-PE, anti-HLA-DR-PE-Cy7, anti-CD45RA-FITC, anti-CD11c-APC, and anti-CD11b-Pacific Blue; Becton Dickinson (BD) Biosciences—Pharmingen, San Diego, CA, USA) and then washed twice with phosphate buffered saline (PBS) containing 1% heat inactivated fetal bovine serum (FBS) (GIBCO). The cells were processed in the FACSCanto II flow cytometer (BD Biosciences, CA, USA) and then analyzed with FlowJo software (TreeStar Inc., Ashland, OR, USA). Cells were gated in a SSC (side scatter)/FSC (forward scatter) plot with the scatter gate for monocytes partially extending into lymphocytes [25]. The cells containing all monocytes and a part of the lymphocyte population were then gated in an HLA-DR/CD14 plot to exclude HLA-DR-negative natural killer cells (which would otherwise contaminate the CD14^+^CD16^++^ subpopulation) and finally were analyzed for CD14 and CD16 expression [25, 26]. Monocyte subsets were defined according to the expression of CD16 (Fc*γ* receptor type III) and CD14 (lipopolysaccharide receptor) as classical monocytes (CD14^++^CD16^−^), intermediate monocytes (CD14^++^CD16^+^), and nonclassical monocytes (CD14^+^CD16^++^) [15]. The expression of *β*_2_-integrins (CD11b and CD11c), CD45RA, and HLA-DR on the monocyte subsets was quantified. The results have been presented as MFI (mean fluorescence intensity). Absolute numbers of monocytes per *μ*L were calculated using peripheral white blood cell counts. In separate experiments, BD Trucount beads were used, confirming that both methods yielded similar absolute values of cell counts.

### 2.5. Intima Media Thickness (IMT)

Intima media thickness of carotid arteries was measured in B-mode presentation using GE Vivid 4 Ultrasound (GE Healthcare, Little, Chalfont, UK) with 10 MHz linear probe according to previously published protocol [27]. IMT was measured bilaterally at the proximal and distal walls 1 cm before bifurcation, at the bifurcation of common carotid arteries, and 1 cm after bifurcation in internal carotid arteries. Measurement was gated with ECG. Analysis was performed off-line using Image ProPlus software (Media Cybenetics Inc., Rockville, MD, USA). The average value from 12 measurements was used in analyses.

## 3. Statistics

Data are reported as mean (SD) or median (interquartile range, IRQ) unless otherwise indicated. Proportions were compared using Chi-square test or Fisher's exact test in case of small samples. Distribution of quantitative variables was tested by Shapiro-Wilk and Kolmogorov-Smirnov tests. To compare measures between the non-RA and RA patients, Student's *t*-test with independent estimation of variances or Mann-Whitney test was used where appropriate.

Generalized Linear Models (GLM) were used to test differences between the three groups: non-RA and RA subjects with low and high disease activity—type III sum of square was used. Homogeneity of variances was verified by Levene's test. The Bonferroni test was performed in post-hoc comparisons. In case of lack of normal distribution Kruskal-Wallis test was used.

Owing to multiple comparisons, two-tailed *P* values less than 0.01 were considered to be statistically significant. Statistical analysis was performed by STATISTICA software v.10 (StatSoft Inc.).

## 4. Results

**Figure 1:**
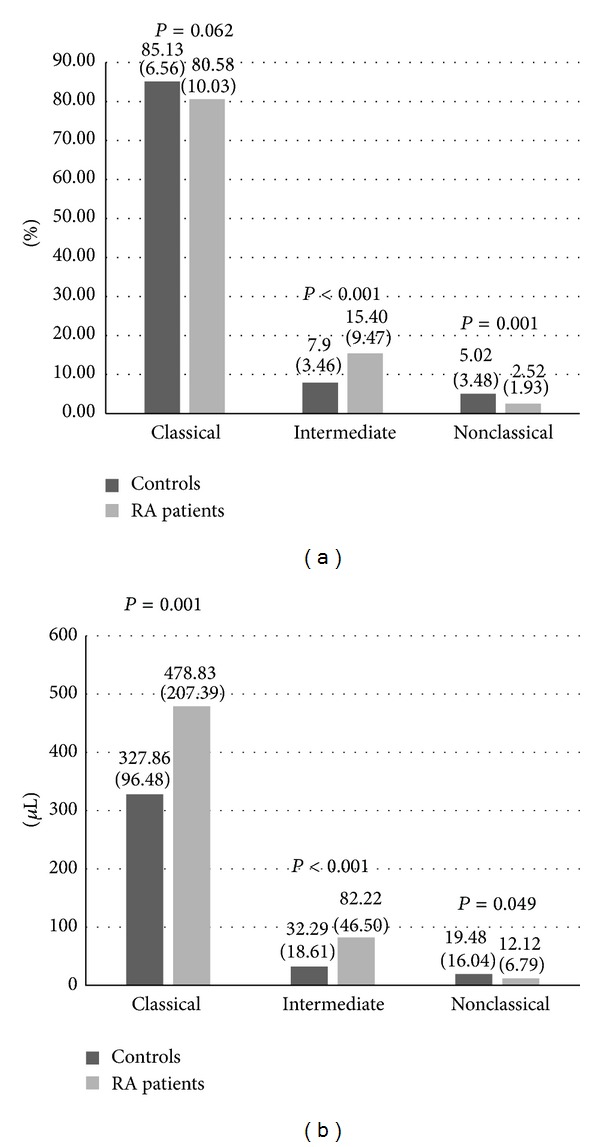
Monocyte subsets in RA patients versus controls. Data are shown as means (SD) in % of monocytes (a) or per *μ*L of blood (b). RA: rheumatoid arthritis.

Comparisons of monocyte subsets, CD45RA, HLA DR, and *β*_2_ integrin expression and markers of cardiovascular risk between RA patients and control subjects are presented in Figure 1 and Tables 1 and 2.

Clinical and biochemical characteristics of RA patients and control subjects in terms of traditional risk factors were comparable. IMT was also comparable between the two groups (Table 1). The mean values of traditional CV risk factors in both groups were within normal range [14], except for mean value of total cholesterol in control group, and LDL cholesterol in both groups, that were marginally elevated. The percentage of patients with elevated levels of particular risk factors was comparable in the study and control group (data not shown). RA patients had increased hsCRP compared to controls.

RA patients had an increased absolute number of monocytes compared to controls. We observed increased number and percentage of intermediate (CD14^++^CD16^+^) monocytes and an increased number of classical (CD14^++^CD16^−^) monocytes and a decreased percentage of nonclassical (CD14^+^CD16^++^) monocytes in RA patients in comparison with healthy subjects (Figure 1). RA patients exhibited higher CD11c and HLA-DR expression on nonclassical (CD14^+^CD16^++^) monocytes and lower CD45RA expression on intermediate (CD14^++^CD16^+^) monocytes. There were no differences in the monocyte CD11b expression in patients when compared to the control group.

**Figure 2:**
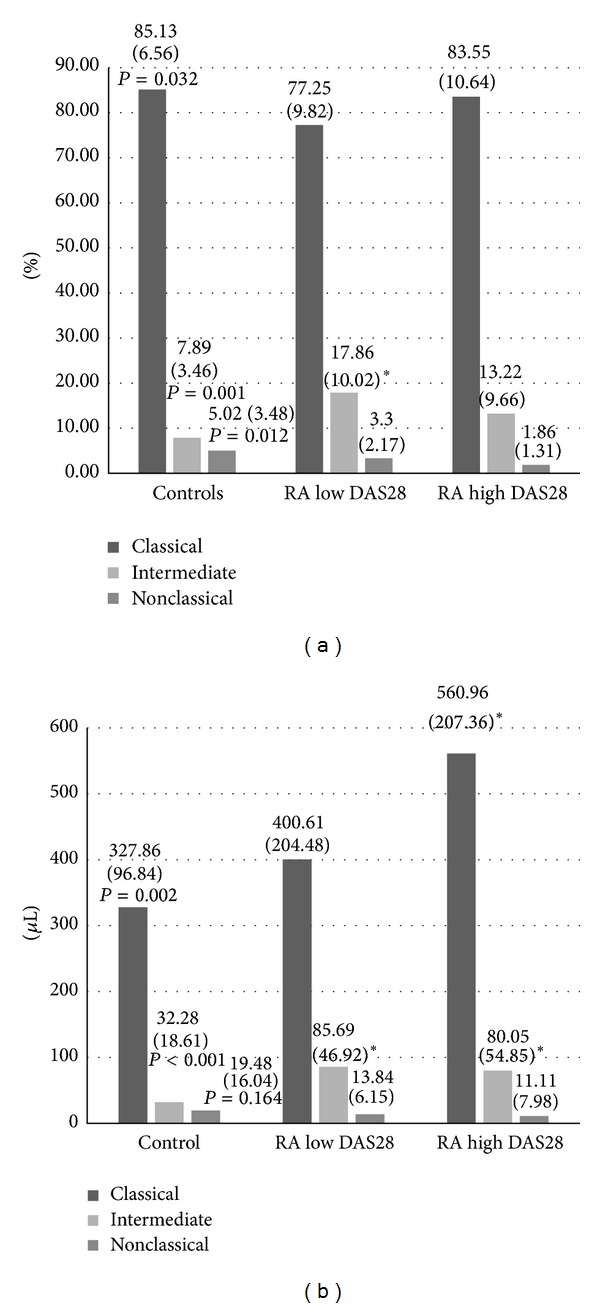
Monocytes subpopulations according to DAS28. Data are shown as means (SD) in % of monocytes (a) or per *μ*L of blood (b). DAS28: disease activity score based on the assessment of 28 joints; low DAS28 = (2.6–5.1); high DAS28 = (>5.1). RA: rheumatoid arthritis; *P* value in ANOVA (GLM models). *P < 0.01 versus control group in post-hoc analyses.

Comparisons of monocyte subsets, CD45RA, HLA DR, *β*_2_ integrin expression, and conventional cardiovascular risk factors in RA patients according to disease activity with the reference to control subjects are presented in Figure 2 and Tables 3 and 4. There were no significant differences between patients with low and high disease activity in numbers and percentages of monocyte subsets. However, in relation to control group, RA patients with low disease activity exhibited an increased number and percentage of intermediate (CD14^++^CD16^+^) monocytes, whereas patients with high disease activity exhibited an increased absolute monocyte number and an increased number of classical (CD14^++^CD16^−^) and intermediate (CD14^++^CD16^+^) monocytes. A number of classical monocytes were slightly increased with higher disease activity, but without statistical significance.

HLA-DR expression on classical (CD14^++^CD16^−^) monocytes was higher in patients with lower disease activity than in those with higher disease activity. A similar relationship was observed for intermediate and nonclassical monocytes, however, without statistical significance. Additionally, compared to control subjects, in patients with lower DAS28, we observed higher HLA-DR expression on classical and nonclassical monocytes.

With regard to traditional risk factors, patients with high disease activity had increased systolic blood pressure and MAP in relation to control subjects (Table 3).

## 5. Discussion

Patients with rheumatoid arthritis of short duration had similar cardiovascular risk profile compared to controls. Intima media thickness was also comparable between RA patients and controls. Contrary to our results, IMT was previously reported to be increased in RA patients with recent disease onset [13], but those patients were older (22–78 years old) and subjects with overt cardiovascular disease were included in the study. In the recently published meta-analysis of 22 studies related to carotid intima media thickness in RA patients IMT was increased in 17 studies in comparison to controls [28]. However, most of the studies involved patients with long-standing disease and neither disease duration nor disease activity but the presence of cardiovascular risk factors had significant influence on IMT differences observed between the groups. In the present study, RA patients did not have subclinical atherosclerosis which might be related to short duration of rheumatoid arthritis and the lack of traditional CV risk factors in this selected group of patients. Although increased incidence of CV events in RA demonstrated in other studies is a consequence of accelerated atherosclerosis [2], it cannot be fully explained by traditional CV risk factors [29, 30]. Accelerated atherosclerosis accompanying RA is linked to endothelial activation [21, 31] and dysfunction [32]. We have previously shown [21] that patients with RA of short duration exhibit endothelial activation (expressed by increased level of soluble sVCAM-1, MCP-1, and von Willebrand factor and pentraxin-3) that is an important factor in the development of atherosclerosis.

We observed increased total monocytes number in RA patients. Monocytosis has been described as an independent marker of risk of stable coronary artery disease and acute myocardial infarction [33]. Heine et al. revealed that intermediate (CD14^++^CD16^+^) monocytes but not total monocyte numbers predict cardiovascular events in dialysis patients [17]. Moreover, Berg et al. showed that classical (CD14^++^CD16^−^) monocytes can predict future CV risk independently of other risk factors in a randomly selected population [34]. Considering these findings, increased levels of both intermediate and classical monocytes, which contributed to elevated total monocytosis in our study, might precede the subclinical changes in the arteries.

Assessing the distribution of monocyte subsets in DMARDs-naïve patients with RA of short duration, we revealed higher percentage and number of intermediate (CD14^++^CD16^+^) monocytes and number of classical (CD14^++^CD16^−^) peripheral blood monocytes and decreased percentage of nonclassical (CD14^+^CD16^++^) monocytes in comparison to the control group. Patients with high and low disease activity had similar numbers and percentages of monocyte subsets. Increased prevalence of CD16^+^ monocytes in RA has previously been reported. Kawanaka et al. reported higher frequency of CD16^+^ monocytes in the peripheral blood of patients with RA [18], but without distinguishing between subpopulations of CD16^+^monocytes. Rossol et al. and Cooper et al., who analyzed three subpopulations of monocytes, revealed in patients with RA an expansion of the intermediate (CD14^++^CD16^+^) monocyte population with no growth within the population of nonclassical (CD14^+^CD16^+^) monocytes [19, 20]. However, studies mentioned above considered mainly patients with long-standing RA. Rossol et al. revealed no correlation between expansion of intermediate (CD14^++^CD16^+^) monocytes and the duration of RA, and intermediate monocytes remained stable for up to 4 years in the longitudinal analysis [19]. Although patients with RA of short duration in our study presented increased levels of intermediate monocytes, it did not reflect their increased functional properties as HLA-DR, CD45RA and CD11c expression in this subpopulation of monocytes was not increased. The expression of CD45RA on intermediate monocytes was significantly lower in RA subjects than in healthy controls. CD45RA is considered a marker of activation of peripheral blood monocytes [5]. It was shown that activation of monocytes* in vitro* induces the expression of CD45RA [35] and especially CD16 positive monocytes exhibit high CD45RA expression [5, 36]. Therefore, rather an increase in CD45RA expression on monocytes in RA patients might be expected. Lower CD45RA expression may result from decreased number of activated cells in peripheral blood due to their migration to inflammatory sites.

An interesting observation in our study is decreased percentage of nonclassical monocytes in peripheral blood, which may result from the migration of this monocyte subset into the joints where they might contribute to synovial inflammation. The previously published study revealed that percentage of CD16^+^monocytes was significantly increased in synovial fluid, nearly four times higher compared to peripheral blood [18]. We found enhanced expression of CD11c on nonclassical (CD14^+^CD16^++^) monocytes in RA patients compared to healthy controls. Ancuta et al. demonstrated that CD16^+^monocytes adhere to activated endothelium and migrate into the joint more efficiently than CD16^−^ monocytes due to increased adhesion molecule and chemokine receptor expression [37, 38]. It has been shown lately that, in patients with early RA [39] when compared to healthy controls, the expression of CD11c was higher in both nonclassical and classical monocyte subpopulations, and further nonclassical monocytes displayed enhanced expression of CD11c compared to classical and intermediate monocyte subpopulations. Additionally, nonclassical (CD14^+^CD16^++^) monocytes in our study, in spite of their lower percentage in RA patients, are characterized by enhanced HLA-DR expression compared to healthy controls, mainly in patients with low disease activity. The upregulation of CD11c and HLA-DR may be related to the state of activation of nonclassical monocytes, their higher antigen presentation capacity and enhanced interaction with endothelial cells. Previously [21], in patients with RA of short duration we showed increased levels of sVCAM-1 and MCP-1, endothelial activation markers associated with monocyte adhesion. VCAM-1, upregulated in endothelial cells in response to cytokines such as TNF-*α* and IL-1, is an endothelial ligand for CD11c/CD18, a *β*_2_-integrin expressed on monocytes [9] and involved in monocytes transendothelial migration [40]. Our results showing increased expression of CD11c on nonclassical monocytes may suggest enhanced interactions with endothelium and their contribution to local inflammation.

We found in our study only increased number of classical (CD14^++^CD16^−^) monocytes in patients with RA of short duration in comparison to control subjects, with comparable percentages of classical monocytes between RA patients and controls. In the study of Cooper et al., in the group of early RA patients, percentages of classical CD14^++^CD16^+^ monocytes were not significantly different from healthy controls, but long-standing RA patients had higher prevalence of classical monocytes than patients with early disease [20]. Classical monocytes are thought to correspond to murine peripheral blood Ly-6C^high^ monocytes, whose number increases in conditions of inflammation and that are recruited into sites of inflammation [41]. Moreover, elevated classical monocytes were shown to predict CV events [34]. In our study, we observed higher expression of HLA-DR on classical monocytes in RA patients with lower disease activity than in those with higher disease activity. It was observed also on other monocyte subsets but without statistical significance. Differential associations of inflammatory and endothelial biomarkers with disease activity in RA of short duration were discussed previously [21]. In the present study, higher HLA-DR expression on classical monocytes, which indicates their increased activation status, was shown despite lower disease activity, which may suggest different mechanisms involved in monocytes activation and in the level of systemic inflammation assessed by disease activity score.

## 6. Study Limitations

There are some limitations to our study. First of all our conclusions are constrained by low number of study participants and a cross-sectional design of the study which makes it impossible to follow changes in the relationship between markers of inflammation, count and activity state of monocytes in the course of disease. Second, we could not eliminate influence of steroids on monocytes and endothelial function in patients with rheumatoid arthritis. Third, the results of the cells surface antigen expression may be affected by the method used for leukocyte isolation.

## 7. Conclusions 

Patients with rheumatoid arthritis of short duration show increased total monocytosis and alteration in peripheral blood monocyte subsets despite the fact that there is no evidence of subclinical atherosclerosis. Disease activity assessed with DAS28 was associated with impaired functional properties of monocytes but not with a shift in monocyte subpopulations. Further studies are needed to establish whether these alterations are relevant to the increased cardiovascular risk in RA.

## Figures and Tables

**Table 1:** Clinical characteristics and cardiovascular (CV) risk factors of RA patients and control subjects.

	RA patients (*n* = 27)	Control group (*n* = 22)	*P* value
	Mean (SD)	Mean (SD)	
Clinical characteristics			
Age, years	41.40 (9.65)	38.13 (10.84)	0.270
Female gender, *n* (%)	21 (77.78%)	17 (77.27%)	1.000
Smoking habit, number (%)	12 (44.44%)	4 (18.18%)	0.069
RF positivity, *n* (%)	25 (86%)	—	NA
aCCP positivity, *n* (%)	24 (92.30%)	—	NA
Disease duration, months (median)	12 [4; 24]	—	NA
DAS28	4.42 (1.50)	—	NA
Steroids, number (%)	10 (37%)	—	NA
NSAIDs, number (%)	19 (70.37%)	—	NA
Without NSAIDs or steroids, number (%)	4 (14.8%)	—	NA
Antihypertensives, number (%)	2 (7.41%)	0	0.490
hsCRP, mg/L	15.92 (25.38)	1.03 (0.89)	**<0.001**
Traditional CV risk factors			
Systolic blood pressure, mmHg	121.77 (18.11)	113.86 (10.99)	0.066
Diastolic blood pressure, mmHg	80.03 (7.95)	75.27 (6.60)	0.041
Mean arterial pressure, mmHg	93.95 (10.50)	88.13 (7.18)	0.032
Body mass index, kg/m^2^	23.36 (4.09)	23.34 (2.72)	0.876
Glucose, mmol/L	4.64 (0.40)	5.02 (0.63)	0.015
TC, mmol/L	5.03 (1.24)	5.20 (0.70)	0.552
LDL-C, mmol/L	3.06 (0.96)	3.05 (0.74)	0.959
HDL-C, mmol/L	1.57 (0.41)	1.73 (0.45)	0.316
Triglycerides, mmol/L	0.94 (0.47)	0.93 (0.37)	0.795
IMT (mm)	0.43 (0.04)	0.48 (0.11)	0.114

Data are shown as means (SD) or medians [interquartile range, IRQ] or percentages (%). NA: not applicable; RA: rheumatoid arthritis; RF: rheumatoid factor; aCCP: anticyclic citrullinated peptide antibodies; DAS28: disease activity score in 28 joints; NSAIDs: nonsteroidal anti-inflammatory drugs; hsCRP: high-sensitivity C-reactive protein; TC: total cholesterol; LDL-C: low-density lipoproteins-cholesterol; HDL-C: high-density lipoproteins-cholesterol; IMT: intima media thickness.

**Table 2:** Monocyte subpopulations and their characteristics (total count, expression of HLA-DR, CD45RA, and *β*_2_-integrins).

	RA (*n* = 27)	Control group (*n* = 22)	*P* value
	Mean (SD)	Mean (SD)	
Monocytes total count (per *μ*L of blood)	581.85 (220.24)	387.14 (110.15)	**<0.001**
CD14^++^CD16^−^ classical monocytes			
CD45RA, MFI	646.92 (182.17)	780.31 (293.99)	0.028
CD11c, MFI	1574.44 (609.28)	1286.57 (1072.39)	0.018
CD11b, MFI	1468.07 (841.15)	1707.85 (1816.07)	0.581
HLA-DR, MFI	7781.88 (3777.45)	6116.86 (2040.72)	0.072
CD14^++^CD16^+^ intermediate monocytes			
CD45RA, MFI	696.59 (199.02)	1079.45 (594.60)	**0.001**
CD11c, MFI	2809.56 (918.04)	2877.95 (2235.30)	0.289
CD11b, MFI	1647.30 (932.19)	1937.57 (2182.40)	0.546
HLA-DR, MFI	26656.89 (8883.37)	30188.32 (7584.79)	0.146
CD14^+^CD16^++^ nonclassical monocytes			
CD45RA, MFI	1942.93 (589.65)	3512.00 (2599.85)	0.029
CD11c, MFI	5038.59 (1981.75)	3532.43 (2599.87)	**0.002**
CD11b, MFI	770.96 (365.94)	855.19 (738.51)	0.692
HLA-DR, MFI	32300.37 (11860.56)	21097.32 (8487.21)	**<0.001**

Data are shown as means (SD). RA: rheumatoid arthritis; MFI: mean fluorescence intensity.

**Table 3:** Traditional cardiovascular risk factors according to DAS28.

	Control group (*n* = 22)	RA patients with low DAS28^∧^ (*n* = 14)	RA patients with high DAS28^∧^ (*n* = 10)	*P* value
	Mean (SD)	Mean (SD)	Mean (SD)	
Age, years	38.13 (10.84)	39.92 (9.29)	43.60 (0.17)	0.385
Female gender, *n* (%)	17 (77.27%)	11 (78.57%)	7 (70%)	0.878^†^
Smoking habit, number (%)	4 (18.18%)	5 (35.71%)	6 (60%)	0.024^†^
Steroids, number (%)	—	3 (21.43%)	4 (40%)	0.616^†^
NSAIDs, number (%)	—	8 (57.14%)	8 (80%)	0.490^†^
hsCRP, mg/L	1.03 (0.89)	5.43 (6.76)∗	35.12 (33.74)∗	**<0.001**
Systolic blood pressure, mmHg	113.86 (10.99)	115.14 (16.87)	132.10 (16.65)∗	**0.004**
Diastolic blood pressure, mmHg	75.27 (6.60)	78.35 (7.65)	82.90 (8.41)∗	0.031
Mean arterial pressure, mmHg	88.13 (7.18)	90.61 (10.02)	99.30 (9.79)∗	**0.006**
Body mass index, kg/m^2^	23.34 (2.72)	23.90 (3.68)	22.76 (4.73)	0.740
Glucose, mmol/L	5.02 (0.63)	4.58 (0.30)	4.80 (0.49)	0.063
TC, mmol/L	5.20 (0.70)	5.22 (1.33)	4.57 (1.16)	0.266
LDL-C, mmol/L	3.05 (0.74)	3.24 (0.99)	2.79 (0.97)	0.529
HDL-C, mmol/L	1.73 (0.45)	1.60 (0.45)	1.46 (0.38)	0.352
Triglycerides, mmol/L	0.93 (0.37)	0.85 (0.31)	0.82 (0.39)	0.726
IMT (mm)	0.48 (0.11)	0.43 (0.05)	0.42 (0.04)	0.208

Data are shown as means (SD). ^∧^Low DAS28 = (2.6–5.1); high DAS28 = (>5.1). RA: rheumatoid arthritis; DAS28: disease activity score in 28 joints; NSAIDs: nonsteroidal anti-inflammatory drugs; hsCRP: high-sensitivity C-reactive protein; TC: total cholesterol; LDL-C: low-density lipoproteins-cholesterol; HDL-C: high-density lipoproteins-cholesterol; IMT: intima media thickness.

*P* value in ANOVA (GLM models). ∗*P* < 0.01 versus control group in post-hoc analyses ^†^ *P*: patients with low disease activity versus patients with high disease activity.

**Table 4:** Monocyte subpopulations and their characteristics (total count, expression of HLA-DR, CD45RA, and *β*_2_-integrins) according to DAS28.

	Control group (*n* = 22)	RA patients with low DAS28^∧^ (*n* = 14)	RA patients with high DAS28^∧^ (*n* = 10)	*P* value
	Mean (SD)	Mean (SD)	Mean (SD)	
Monocytes total count (per *μ*L of blood)	387.14 (110.15)	508.57 (240.63)	661.00 (194.90)∗	**0.001**
CD14^++^CD16^−^ classical monocytes				
CD45RA, MFI	780.30 (293.99)	621.00 (132.48)	696.70 (250.78)	0.174
CD11c, MFI	1286.57 (1072.39)	1448.64 (527.74)	1662.20 (722.06)	0.526
CD11b, MFI	1707.85 (1816.07)	1281.42 (684.76)	1644.80 (1087.00)	0.665
HLA-DR, MFI	6116.86 (2040.72)	9559.00 (4229.12)∗	5628.70 (2141.07)^#^	**0.001**
CD14^++^CD16^+^ intermediate monocytes				
CD45RA, MFI	1079.45 (594.60)	680.71 (161.28)	756.80 (253.02)	0.023
CD11c, MFI	2877.95 (2235.30)	2645.50 (874.77)	2886.20 (1051.87)	0.910
CD11b, MFI	1937.57 (2182.40)	1428.57 (786.51)	1798.60 (1134.00)	0.670
HLA-DR, MFI	30188.32 (7584.79)	31106.93 (8237.30)	21746.10 (8066.17)	0.011
CD14^+^CD16^++^ nonclassical monocytes				
CD45RA, MFI	3512.00 (2599.85)	1764.64 (405.46)	2281.00 (713.70)	0.157
CD11c, MFI	3532.42 (2599.85)	4235.21 (1422.03)	5655.50 (2402.56)	0.060
CD11b, MFI	855.19 (738.51)	696.64 (368.87)	762.00 (317.71)	0.715
HLA-DR, MFI	21097.32 (8487.21)	35553.21 (11818.02)∗	28167.20 (11481.66)	**<0.001**

Data are shown as means (SD). ^∧^Low DAS28 = (2.6–5.1); high DAS28 = (>5.1). RA: rheumatoid arthritis; MFI: mean fluorescence intensity.

*P* value in ANOVA (GLM models). ∗*P* < 0.01 versus control group, ^#^ *P* < 0.01 versus RA patients with low disease activity in post-hoc analyses.
